# Comparison of Japanese Mpox (Monkeypox) Health Education Materials and Texts Created by Artificial Intelligence: Cross-Sectional Quantitative Content Analysis Study

**DOI:** 10.2196/70604

**Published:** 2025-10-17

**Authors:** Shinya Ito, Emi Furukawa, Tsuyoshi Okuhara, Hiroko Okada, Takahiro Kiuchi

**Affiliations:** 1 School of Nursing Kitasato University Sagamihara Japan; 2 University Hospital Medical Information Network Center The University of Tokyo Hospital Tokyo Japan; 3 Department of Health Communication, School of Public Health Graduate School of Medicine The University of Tokyo Tokyo Japan

**Keywords:** mpox, patient education, AI-generated health education, readability, Japan, health communication, artificial intelligence

## Abstract

**Background:**

Mpox (monkeypox) outbreaks since 2022 have emphasized the importance of accessible health education materials. However, many Japanese online resources on mpox are difficult to understand, creating barriers for public health communication. Recent advances in artificial intelligence (AI) such as ChatGPT-4o show promise in generating more comprehensible and actionable health education content.

**Objective:**

The aim of this study was to evaluate the comprehensibility, actionability, and readability of Japanese health education materials on mpox compared with texts generated by ChatGPT-4o.

**Methods:**

A cross-sectional study was conducted using systematic quantitative content analysis. A total of 119 publicly available Japanese health education materials on mpox were compared with 30 texts generated by ChatGPT-4o. Websites containing videos, social media posts, academic papers, and non-Japanese language content were excluded. For generating ChatGPT-4o texts, we used 3 separate prompts with 3 different keywords. For each keyword, text generation was repeated 10 times, with prompt history deleted each time to prevent previous outputs from influencing subsequent generations and to account for output variability. The Patient Education Materials Assessment Tool for Printable Materials (PEMAT-P) was used to assess the understandability and actionability of the generated text, while the Japanese Readability Measurement System (jReadability) was used to evaluate readability. The Journal of the American Medical Association benchmark criteria were applied to evaluate the quality of the materials.

**Results:**

A total of 119 Japanese mpox-related health education web pages and 30 ChatGPT-4o–generated texts were analyzed. AI-generated texts significantly outperformed web pages in understandability, with 80% (24/30) scoring ≥70% in PEMAT-P (*P*<.001). Readability scores for AI texts (mean 2.9, SD 0.4) were also higher than those for web pages (mean 2.4, SD 1.0; *P*=.009). However, web pages included more visual aids and actionable guidance such as practical instructions, which were largely absent in AI-generated content. Government agencies authored 90 (75.6%) out of 119 web pages, but only 31 (26.1%) included proper attribution. Most web pages (117/119, 98.3%) disclosed sponsorship and ownership.

**Conclusions:**

AI-generated texts were easier to understand and read than traditional web-based materials. However, web-based texts provided more visual aids and practical guidance. Combining AI-generated texts with traditional web-based materials may enhance the effectiveness of health education materials and improve accessibility to a broader audience. Further research is needed to explore the integration of AI-generated content into public health communication strategies and policies to optimize information delivery during health crises such as the mpox outbreak.

## Introduction

Since May 2022, outbreaks of mpox (formerly known as monkeypox) have been reported primarily in Europe and the United States, with more than 99,500 cases and more than 200 deaths reported from 122 countries [1]. Japan has reported 247 cases and 1 death [1]. Outbreaks of mpox appear to occur disproportionately among men who have had sexual contact with other men [2], but infection can also occur through close and prolonged physical contact, regardless of sex, gender identity, or sexual orientation [3].

The increasing availability of health education materials online has transformed public health education, particularly regarding topics related to sexual health, where accessibility and anonymity are important [4]. However, many patient education materials created by medical professionals are written at a reading level higher than that recommended for the general adult population, thereby creating a barrier to understanding critical health information [5-10]. The National Workgroup on Cancer and Health [11], the American Medical Association [12], and the Centers for Disease Control and Prevention [13] recommend that patient educational texts be readable at a sixth- to eighth-grade level or lower. However, evaluations of mpox-related content on the internet to date have indicated that much of this material is difficult for the general public to understand [14].

Artificial intelligence (AI) tools such as ChatGPT offer novel approaches to making health education materials more accessible. Recent studies have shown that AI-generated texts can simplify complex medical information and improve readability, outperforming traditional web-based materials [14-16]. By enhancing clarity and accessibility, AI-generated texts have the potential to reduce health disparities and ensure equitable access to vital health information, particularly during public health crises such as the mpox outbreak.

This study aims to evaluate the comprehensibility, actionability, and readability of Japanese online health education materials on mpox in comparison with content generated by ChatGPT-4o. Our findings may guide public health policies by demonstrating how AI tools can improve the clarity of health education materials. Further, this research will support the integration of AI-generated text into public health strategies, potentially reducing the burden on health care professionals and enhancing communication during emergencies.

## Methods

### Information Generation and Website Selection

We conducted a systematic quantitative content analysis of online texts adopting a cross-sectional design. From August 20 to August 30, 2024, we searched for web pages by using the Google search engine. Concurrently, texts were generated using ChatGPT-4o. We used the Google search engine to identify web pages by entering the terms “mpox” (in English), “エムポックス” (Japanese phonetic transcription), and “サル痘” (the traditional Japanese term for monkeypox). From the top-ranking results, we excluded videos, social networking services, expert websites, academic papers, subscription-based or paywalled sites, non-Japanese language websites, and pages that only listed URLs. We identified 119 web pages for analysis.

For ChatGPT-4o, we used the prompt “Please tell me about [keyword],” entering each of the 3 keywords (mpox, エムポックス, サル痘) separately. For each keyword, text generation was repeated 10 times, with prompt history deleted each time to prevent previous outputs from influencing subsequent generations and to account for output variability. This methodological design was intended to reflect realistic information-seeking behavior among general users, particularly those with limited prior knowledge of mpox, who are likely to initiate searches by using only simple keyword terms.

An epidemiology researcher independently scored all the documents. To ensure validation, we randomly selected 20% (30/150) of the documents for independent scoring by a physician. Each document was first assigned a unique ID, and Microsoft Excel’s RAND() function was used to generate a random number for each entry. The documents were then sorted by a random number, and the top 20% were selected. A physician then scored the selected subset by using the Japanese version of Patient Education Materials Assessment Tool for Printable Materials (PEMAT-P) [17,18]. In cases of disagreement, consensus was reached through discussion.

### Ethical Considerations

This study was not reviewed by an ethics board because no human participants or interventions were involved.

### Understandability and Actionability

The ease of understandability and actionability of the text on the website and the text generated by ChatGPT-4o were evaluated using the Japanese version of PEMAT-P [17,18]. The Japanese version of PEMAT-P comprises 23 items, and evaluates content, word choice and style, use of numbers, structure, layout and design, and use of visual materials. The final score for PEMAT-P ranges from 0% to 100%, with a higher score indicating that the text is easier to understand and easier to act on. The cutoff score for both ease of understanding and actionability is 70%.

### Readability

In this study, the Japanese Readability Measurement System (jReadability) was used to quantitatively evaluate the readability of Japanese texts [19,20]. This measurement system calculates the difficulty of Japanese based on the average sentence length, word difficulty, grammatical part-of-speech ratio, and character type of each text. A higher score indicates that the text is relatively easy to read: 0.5-1.4, very difficult to read; 1.5-2.4, difficult to read; 2.5-3.4, somewhat difficult to read; 3.5-4.4, neither difficult nor easy to read; 4.5-5.4, easy to read; and 5.5-6.4, very easy to read. For example, “very difficult” refers to a level where the skills required allow one to understand even highly specialized texts.

### Credibility

To assess the reliability of texts on web pages, we used the benchmark criteria outlined by the Journal of the American Medical Association [21]. These criteria consist of authorship (authors and contributors with affiliations and relevant qualifications), attribution (references and sources with copyright information disclosed), timeliness (date of content publication and revision dates clearly stated), and disclosure (disclosure of site ownership along with sponsorship, advertising, warranties, and financial support).

### Statistical Analyses

Statistical analyses were conducted using 2-sided *t* tests, chi-square tests, and Fisher exact tests to compare sentences from web pages with those generated by ChatGPT-4o. In addition, intraclass correlation coefficients (ICCs) were calculated to assess interrater reliability. A significance level of *P*<.05 was applied for all statistical tests. Data analysis was performed using SPSS Statistics for Windows (version 25.0; IBM Corp).

## Results

### Information Sources

Analysis was performed for 119 web pages from Google and 30 sentences generated by ChatGPT-4o. None of the sentences generated by ChatGPT-4o contained any obvious errors. The ICC was calculated to quantify the degree of agreement between items, with ICC=0.43 for PEMAT-P understandability and ICC=0.43 for actionability. The characteristics of the web pages are summarized in Table 1. For Japanese mpox-related web pages, those generated by the government were the most common (90/119, 75.6%). Web pages were last updated after 2021, with 51 (42.9%) pages updated in 2023 and 40 (33.6%) pages updated in 2024. For the JAMA benchmark criteria, 117 (98.3%) pages had disclosure, but only 31 (26.1%) had attribution.

**Table 1:** Characteristics of the web page search results (N=119).

		Values, n (%)
**Source**		
	Government	90 (75.6)
	Business	15 (12.6)
	Academic organization	6 (5.0)
	Nonprofit organization	5 (4.2)
	Medical organization	3 (2.5)
**Year of website update**		
	2021	2 (1.7)
	2022	13 (10.9)
	2023	51 (42.9)
	2024	40 (33.6)
	Unknown	13 (10.9)
**Journal of the American Medical Association benchmark criteria**		
	Authorship	93 (78.2)
	Attribution	31 (26.1)
	Disclosure	117 (98.3)
	Currency	105 (88.2)

### Understandability and Actionability

**Figure 1:**
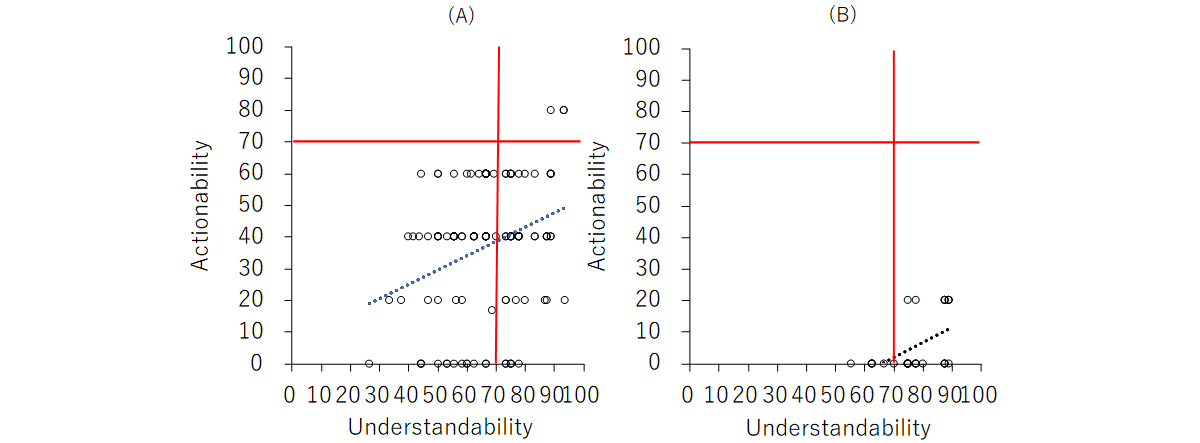
Patient Education Materials Assessment Tool for Printable Materials evaluation of understandability and actionability of web search (A) and ChatGPT-4o–generated texts (B). The red lines indicate a 70% threshold for both dimensions. Dots represent individual texts, and dashed lines represent regression trends.

The results of the comparisons between web page texts and sentences generated by ChatGPT-4o are shown in Table 2 and Figure 1. The proportion of PEMAT-P understandability scores ≥70% (as defined in the Methods section) was significantly higher for ChatGPT-4o texts (24/30, 80%; *P*<.001). Mean (SD) PEMAT-P scale scores for texts from ChatGPT-4o were significantly higher for understandability (77.1, SD 9.7; mean difference –10.3, 95% CI –14.7 to –5.9; *P*<.001) and lower for actionability (5.3, SD 9.0; mean difference 31.9, 95% CI 26.9-37.0; *P*<.001) than for texts from web pages.

**Table 2:** Comparison of the item scores between artificial intelligence chatbots and web search–generated texts.

		Web search (n=119)	ChatGPT-4o (n=30)	Test value *(df)*	*P* value
**Patient Education Materials Assessment Tool for Printable Materials, n (%)**					
	Understandability (≥70)	51 (42.9)	24 (80.0)	13.2 (1.0)^a^	<.001
	Actionability (≥70)	3 (2.5)	0 (0)	0.8 (1.0)^a^	>.99
**Patient Education Materials Assessment Tool for Printable Materials** **score, mean (SD)**					
	Understandability	66.8 (14.2)	77.1 (9.7)	–4.68 (64.4)^b^	<.001
	Actionability	37.3 (21.5)	5.3 (9.0)	12.5 (114.3)^b^	<.001
**jReadability^c^ difficulty level, n (%)**				20.4 (4.0)^a^	<.001
	Very readable	0 (0)	0 (0)		
	Readable	0 (0)	0 (0)		
	Neutral	0 (0)	3 (10.0)		
	Somewhat difficult	60 (50.4)	22 (73.3)		
	Difficult	55 (46.2)	5 (16.7)		
	Very difficult	3 (2.5)	0 (0)		
	Not measurable (too difficult)	1 (0.8)	0 (0)		
jReadability score, mean (SD)		2.4 (1.0)	2.9 (0.4)	–2.6 (147.0)^b^	.009

^a^Chi-square value.

^b^2-sided *t* test value.

^c^jReadability: Japanese Readability Measurement System.

Table 3 shows the results for comparisons of PEMAT-P items. Compared to that in ChatGPT-4o texts, the proportion of web pages that used visual materials (item 12; 25/119, 21%) and the proportion of items related to actionability (items 17-19; *P*<.001) were higher. Conversely, ChatGPT-4o texts were more likely to have a clear purpose (item 1; 30/30, 100%) and to focus on important points by using visual cues (item 11; 25/30, 83%; *P*=.02) than web page texts (*P*=.005).

**Table 3:** Patient Education Materials Assessment Tool for Printable Materials item scores for web search and ChatGPT-4o–generated texts.

			Web search (n=119), n (%)	ChatGPT-4o (n=30), n (%)	Chi-square *(df)*	*P* value
**Understandability**						
	**Content**					
		Item 1: The material makes its purpose completely evident	73 (61.3)	30 (100)	16.8 (1.0)	<.001
		Item 2: The material does not include information or content that distracts from its purpose	118 (99.2)	30 (100)	0.3 (1.0)	>.99
	**Word choice and style**					
		Item 3: The material uses common, everyday language	75 (63.0)	21 (70.0)	0.5 (1.0)	.529
		Item 4: Medical terms are used only to familiarize audience with the terms. When used, medical terms are defined	77 (64.7)	22 (73.3)	0.8 (1.0)	.517
	**Use of numbers**					
		Item 5: Numbers appearing in the material are clear and easy to understand^a^	59 (59.6)	11 (91.7)	4.7 (1.0)	.053
		Item 6: The material does not expect the user to perform calculations^a^	113 (95)	29 (96.7)	0.2 (1.0)	>.99
	**Organization**					
		Item 7: The material breaks or “chunks” information into short section	24 (45.3)	0 (0)	—^b^	—
		Item 8: The material's sections have informative headers	46 (86.8)	0 (0)	—	—
		Item 9: The material presents information in a logical sequence	108 (90.8)	28 (93.3)	0.2 (1.0)	>.99
		Item 10: The material provides a summary	18 (34.0)	0 (0)	1.0 (1.0)	>.99
	**Layout and design**					
		Item 11: The material uses visual cues to draw attention to key points	70 (58.8)	25 (83.3)	6.2 (1.0)	.018
	**Use of visual aids**					
		Item 12: The material uses visual aids whenever they could make content more easily understood	25 (21.0)	0 (0)	7.6 (1.0)	.005
		Item 13: The material's visual aids reinforce rather than distract from the content	31 (83.8)	0 (0)	—	—
		Item 14: The material's visual aids have clear titles or captions	17 (45.9)	0 (0)	—	—
		Item 15: The material uses illustrations and photographs that are clear and uncluttered	23 (62.2)	0 (0)	—	—
		Item 16: The material uses simple tables with short and clear row and column headings^a^	6 (75.0)	0 (0)	—	—
**Actionability**						
	Item 17: The material clearly identifies at least one action the user can take		99 (83.2)	8 (26.7)	37.8 (1.0)	<.001
	Item 18: The material addresses the user directly when describing actions		79 (66.4)	0 (0)	42.4 (1.0)	<.001
	Item 19: The material breaks down any action into manageable, explicit steps		41 (34.5)	0 (0)	14.3 (1.0)	<.001
	Item 20: The material provides a tangible tool whenever it could help the user take action		2 (1.7)	0 (0)	0.5 (1.0)	>.99
	Item 21: The material provides simple instructions or examples of how to perform calculations^a^		—	—	—	—
	Item 22: The material explains how to use the charts, graphs, tables, or diagrams to take actions^a^		1 (0.8)	0 (0)	—	—
	Item 23: The material uses visual aids whenever they could make it easier to act on the instructions		1 (0.8)	0 (0)	0.3 (1.0)	>.99

^a^Including not applicable to the response method.

^b^Not applicable.

### Readability

Regarding the results for jReadability, the most common results for web pages were somewhat difficult (60/119, 50.4%) or difficult (55/119, 46.2%), while the most common result for ChatGPT-4o was somewhat difficult (22/30, 73%). The mean readability score for web page texts (2.4, SD 1.0) was significantly lower than that for ChatGPT-4o–generated texts (2.9, SD 0.4; mean difference –0.5, 95% CI –0.8 to –0.1; *P*=.009), while the difficulty level was significantly higher (*P*=.009). According to the jReadability classification system, the mean score for ChatGPT-4o–generated texts (2.9) corresponds to the “somewhat difficult to read” category, while the mean score for web page texts (2.4) falls into the “difficult to read” category. “Difficult to read” texts typically include complex or technical content, whereas “somewhat difficult to read” texts are generally understandable even if they are slightly technical, especially in everyday contexts.

## Discussion

This study compares the comprehensibility, actionability, and readability of Japanese health education materials on mpox published online with that of AI-generated content from ChatGPT-4o. Our results suggest that AI-generated texts are generally easier to understand and written in simpler Japanese compared to traditional web-based materials. These findings are consistent with those reported in previous research [14,22,23].

AI-generated texts such as those from ChatGPT-4o offer the advantage of providing clearer and more straightforward information, which is critical in the context of public health emergencies. By reducing the cognitive load required to comprehend and act on health information, AI has the potential to improve health literacy and facilitate more effective communication with populations that may face barriers in accessing or understanding complex medical information. However, one key limitation of AI-generated content is the lack of visual aids, charts, and graphs that often accompany web-based texts, which can significantly enhance understanding. Incorporating multimedia elements such as images, diagrams, and graphs into AI-generated content could further improve the comprehensibility and practical application of health information, particularly for complex medical topics.

Regarding actionability, the results showed that web-based texts provided more explicit calls to action than AI-generated content. This difference may be due to the nature of the prompt used to generate AI content, which did not specifically encourage behavior-specific guidance. Research suggests that customized prompts such as asking AI to explain at a sixth-grade level can enhance clarity and actionability [23]. In this study, however, we intentionally used simple keyword-based prompts (eg, Tell me about mpox) to reflect realistic information-seeking behavior by general users. Given that the public understanding of mpox remains limited, particularly in Japan, we assumed that users would initiate queries with minimal phrasing. Assessing the quality of AI-generated materials in response to such basic prompts is important for evaluating the real-world utility of AI in public health education. To further support behavior-oriented communication, developing prompts that elicit more specific and actionable outputs warrants consideration. For instance, prompts such as “Explain mpox to someone with no medical background” or “List 3 actions I should take to avoid mpox” might enhance comprehensibility and actionability. Moreover, instead of expecting complete guidance in a single interaction, users could issue follow-up prompts like “What should I do if I think I might have mpox?” or “How can I talk to my partner about mpox prevention?” These examples are exploratory and suggest that more systematic investigation into prompt design will be essential for maximizing the practical utility of AI-generated health content. For AI-generated health information to be practically applicable and useful in public health contexts, future research should emphasize optimizing prompt use to ensure that the results not only provide information but also encourage preventive actions and healthy behaviors.

The readability analysis suggested that AI-generated texts were easier to read than web-based materials. This finding is important because readability plays a critical role in how effectively public health information can be understood and acted upon, particularly in times of crisis like the mpox outbreak. By maintaining lower levels of text complexity, AI-generated content can serve as a useful tool for delivering health education to broader audiences, including underserved populations.

This study is, to the best of our knowledge, the first to compare health education texts on mpox available on web pages and that available via AI chatbots. This is also the first study to evaluate the understandability, actionability, and writing difficulty of Japanese health education texts on mpox published online. Many searches for health education information related to mpox are likely conducted on the web and via AI chatbots because of ease of access and anonymity. For this reason, we believe evaluating the quality of information related to mpox is important.

Several limitations to this study must be kept in mind. First, AI chatbots sometimes generate inaccurate information; therefore, issues with the reliability of the resulting information need to be investigated. Second, this study used a simple prompt, “Please tell me about the keyword,” and did not consider prompts aimed at reducing sentence difficulty, generating summaries, generating charts, or promoting prevention or health behaviors. Future research should consider prompts that increase comprehension and ease of action while reducing text difficulty. Third, because this was a cross-sectional study, only information available at the time of the survey was assessed. Web and ChatGPT-4o health education texts are constantly changing. For example, as the spread of mpox has increased, information available on the internet has been updated frequently. In addition, new versions of AI chatbots are released every few months, and the quality of the generated text has reportedly improved with each new version [23,24]. In this study, we used ChatGPT-4o (OpenAI) from August 20 to 30, 2024. Therefore, the findings reflect the performance of that specific version at that time. It is important to note that the results of this study merely represent a snapshot of the situation at the time of the evaluation. Additionally, the moderate interrater reliability observed (ICC=0.43) may be explained by differing interpretations between the physician and epidemiologist who served as raters. For instance, in PEMAT item 4 (definition of medical terms), the physician assessed that definitions were present, whereas the epidemiologist judged that the explanations were insufficient due to the complexity of the terminology. In contrast, for item 21 (clear steps for actions), the epidemiologist found the materials actionable, while the physician considered the instructions inadequate. These discrepancies suggest that the raters’ professional backgrounds influenced their assessments, highlighting a potential limitation in applying PEMAT across multidisciplinary evaluators. Finally, it should be noted that current AI models such as ChatGPT-4o cannot generate visual materials (eg, diagrams, illustrations) in response to general prompts such as “Tell me about mpox.” Due to copyright and security limitations, they are also unable to automatically incorporate images from the internet. Therefore, for AI-generated materials to include visual aids, additional steps such as human editing or integration with external tools are required. Alternatively, providing trustworthy visuals from academic articles or official sources to the AI model and requesting explanations based on them may be a practical workaround in future applications.

AI-generated health education materials such as those from ChatGPT-4o appear easier to understand and read compared to traditional web-based content. However, visual aids and clear action-oriented guidance are lacking. These findings suggest that AI can improve how health information is shared with the public, particularly during health emergencies like the mpox outbreak. Nonetheless, more refinement is needed to ensure that the information provided is both actionable and comprehensive. One possible approach to addressing this limitation is prompt engineering, which involves designing questions that elicit more specific, behavior-oriented responses from AI. Additionally, fine-tuning the AI model using public health domain–specific data could help generate more tailored and context-appropriate health guidance.

## Appendix

Multimedia Appendix 1 Complete ChatGPT-4o (OpenAI) conversation transcripts used to generate and evaluate Japanese mpox health education texts.

## Abbreviations

AI: artificial intelligence
ICC: intraclass correlation coefficient
jReadability: Japanese Readability Measurement System
PEMAT-P: Patient Education Materials Assessment Tool for Printable Materials

## Data Availability

The datasets generated and/or analyzed in this study are available from the corresponding author on reasonable request.
