# Supplementary material for: Comparison of Japanese Mpox (Monkeypox) Health Education Materials and Texts Created by Artificial Intelligence: Cross-Sectional Quantitative Content Analysis Study
Source: JMIR AI. 2025 Oct 17;4:e70604. doi: 10.2196/70604 (PMC12579291; doi:10.2196/70604)
Supplement: Multimedia Appendix 1 [file ai_v4i1e70604_app1.docx]

| Mpoxについて教えてください。 | Mpox（旧称：サル痘）は、サル痘ウイルス（Monkeypox virus）によって引き起こされるウイルス性疾患です。主に中央アフリカおよび西アフリカの特定の地域で発生していましたが、近年では他の地域でも報告が増えています。  ### 症状 Mpoxの症状は、天然痘に似ていますが、通常は軽度です。主な症状は以下の通りです：  - **発熱**：通常、感染後6〜13日で発症しますが、5〜21日かかる場合もあります。 - **頭痛**：強い頭痛が伴います。 - **筋肉痛**：全身の筋肉や関節が痛むことがあります。 - **リンパ節腫脹**：特に首、脇の下、鼠径部のリンパ節が腫れることが特徴です。 - **発疹**：顔から始まり、その後体の他の部分に広がる水疱状の発疹が現れます。  ### 感染経路 Mpoxは、感染した動物（特に齧歯類や霊長類）との接触や、感染者との密接な接触を通じて人に伝染します。また、ウイルスは傷口、粘膜、呼吸器系を通じて体内に侵入することがあり、飛沫感染も可能性があります。  ### 予防と治療 - **予防**：ワクチン接種が効果的です。特に天然痘ワクチンがサル痘に対しても予防効果があるとされています。感染地域への渡航時には、動物との接触を避けることが重要です。 - **治療**：特異的な治療法はありませんが、症状を緩和するための対症療法が行われます。また、重症例に対しては抗ウイルス薬が使用されることがあります。  ### 最近の動向 2022年には、Mpoxの感染が従来の流行地域以外でも報告され、国際的な関心が高まりました。これにより、ワクチン接種や監視体制の強化が行われています。  以上がMpoxについての概要です。特定の質問があれば、お答えします。 |
| --- | --- |
| Mpoxについて教えてください。 | Mpox（エムポックス）は、旧称モンキーポックス（Monkeypox）として知られるウイルス性の感染症です。モンキーポックスウイルスによって引き起こされ、通常は西アフリカや中央アフリカの一部の地域で発生しますが、2022年には世界的に広がり、注目を集めました。  ### 症状 Mpoxの症状は、感染後6～13日程度で現れることが多いですが、5～21日の範囲内で変動することもあります。主な症状には以下が含まれます：  - 発熱 - 頭痛 - 筋肉痛 - リンパ節の腫れ（この点が天然痘とは異なります） - 倦怠感 - 皮膚に現れる発疹や水疱（顔、手、足、体に広がることが多い）  発疹は、最初は平らな赤い斑点として現れ、やがて水疱や膿疱に進展し、最後にはかさぶたになって剥がれ落ちます。  ### 感染経路 モンキーポックスウイルスは、感染者や動物（特にサルやげっ歯類）の血液、体液、皮膚病変といったものとの直接接触や、ウイルスを含む飛沫によって感染します。また、汚染された寝具や衣類との接触も感染リスクを高めます。  ### 治療と予防 現在、Mpoxに対して特異的な治療法はなく、症状の緩和が主な治療となります。ただし、天然痘ワクチンがモンキーポックスに対しても一定の予防効果を持つことが示されています。最近では、特定の抗ウイルス薬が治療に使用されることもあります。  ### 公衆衛生対策 2022年の世界的な感染拡大を受けて、世界保健機関（WHO）は対応策を強化し、感染の封じ込めを目指しています。早期診断、接触者追跡、ワクチン接種、感染予防策の徹底などが推奨されています。  Mpoxは、過去には主にアフリカの限られた地域で発生していましたが、世界的な健康問題として認識されるようになり、監視と予防が重要視されています。 |
| Mpoxについて教えてください。 | Mpox（旧称: サル痘）は、サル痘ウイルスによって引き起こされるウイルス性疾患です。サル痘は、もともとアフリカの一部地域で流行していましたが、最近では他の地域でも発生が確認されています。  ### サル痘の特徴: 1. **症状**: サル痘の主な症状には、発熱、頭痛、筋肉痛、リンパ節の腫れ、倦怠感が含まれます。数日後には、発疹が顔や体に現れ、膿疱（膿が溜まった小さな腫れ）に進行します。発疹はかさぶたになり、数週間で治癒します。  2. **感染経路**: サル痘ウイルスは、動物（特に齧歯類や霊長類）から人へ、または人から人へと感染します。感染は、体液や病変に触れること、またはウイルスが含まれる飛沫を吸い込むことによって起こります。  3. **治療**: サル痘には特定の治療法がなく、症状の軽減を目的とした支持療法が行われます。しかし、サル痘ワクチンが存在し、一部のケースでは使用されています。また、サル痘に対しては、痘瘡ワクチンがある程度効果があるとされています。  4. **予防**: サル痘の予防には、感染した動物や人との接触を避けることが重要です。また、サル痘ワクチンの接種が有効な予防策とされています。  5. **最近の状況**: 2022年には、アフリカ以外の国々でもサル痘のアウトブレイクが報告され、世界的な注目を集めました。これにより、サル痘についての認識と予防策が改めて強調されています。  Mpoxは特に新興感染症として注目されており、感染拡大のリスクがあるため、感染予防と早期発見が重要視されています。 |
| Mpoxについて教えてください。 | Mpox（モンキーポックス、旧称：サル痘）は、ポックスウイルス科に属するモンキーポックスウイルスによって引き起こされる感染症です。最初に1958年にサルの間で発見され、1970年に人間においても確認されました。モンキーポックスウイルスは、天然痘ウイルスと関連しており、症状や伝播方法にいくつかの類似点があります。  ### 主な症状 - **発熱**: 初期症状として高熱が現れることが多いです。 - **頭痛や筋肉痛**: 症状が進むにつれて、頭痛や筋肉痛、疲労感が見られます。 - **リンパ節腫脹**: リンパ節が腫れることが一般的で、これが他の類似疾患との識別に役立ちます。 - **発疹**: 数日後に発疹が現れ、顔、手、足から全身に広がることがあります。発疹は水疱状になり、最終的にはかさぶたを形成します。  ### 伝播経路 - **動物から人への感染**: 感染した動物（通常はげっ歯類や霊長類）との接触によって伝播します。咬傷や傷口へのウイルスの侵入が主な感染経路です。 - **人から人への感染**: 飛沫や体液、発疹の内容物との接触、または汚染された物品（衣服、寝具など）を介して感染することがあります。  ### 潜伏期間 潜伏期間は通常6～13日ですが、5～21日の範囲内で変動することがあります。  ### 治療と予防 特定の治療法はありませんが、症状に対する支持療法が行われます。天然痘ワクチンはモンキーポックスに対しても効果があるとされています。また、感染拡大を防ぐための衛生対策や隔離が重要です。  ### 最近の流行 2022年以降、モンキーポックスの症例が増加し、特に欧米での流行が報告されています。このため、世界保健機関（WHO）はモンキーポックスの監視と対応を強化しています。  モンキーポックスは、通常は致死率が低いものの、免疫力が低下している人や子供にとっては重症化する可能性があります。感染症の拡大を防ぐため、早期の診断と適切な医療が求められています。 |
| Mpoxについて教えてください。 | Mpox（旧称: サル痘）は、サル痘ウイルス（Monkeypox virus）によって引き起こされる感染症です。このウイルスは、ポックスウイルス科オルソポックスウイルス属に属し、天然痘ウイルスと非常に近縁です。Mpoxは主にアフリカの中央部および西部の熱帯雨林地域で見られますが、最近では世界中で報告されています。  ### 感染経路 Mpoxは、動物から人への感染と、人から人への感染の両方で広がります。感染源としては、主に感染した動物（特にサル、リス、ネズミなどの齧歯動物）の血液、体液、皮膚病変との接触があります。また、人から人への感染は、主に感染者との密接な接触、呼吸器飛沫、または感染者の皮膚病変との直接的な接触を通じて起こります。  ### 症状 Mpoxの症状は、通常、発熱、頭痛、筋肉痛、リンパ節の腫れなどの全身症状から始まり、その後、皮膚に発疹が出現します。発疹は、顔、手のひら、足の裏、そして体全体に広がり、水疱や膿疱を形成し、最終的にはかさぶたになって治癒します。症状は通常2～4週間続きます。  ### 治療と予防 現在、Mpoxに対する特定の治療法はありませんが、症状を管理するための対症療法が行われます。天然痘に対するワクチンがMpoxにもある程度の予防効果を持つとされています。感染リスクのある人々に対しては、ワクチン接種が推奨されることがあります。  ### 公衆衛生への影響 Mpoxは通常、致死率が比較的低いですが、特定の集団（免疫不全の人々や小児など）では重症化する可能性があります。近年の流行では、都市部や国際的な移動を通じて、かつて見られなかった地域にも拡散するケースが増加しており、公衆衛生上の懸念が高まっています。  これらの情報を基に、Mpoxについてさらに詳しく調査や研究を進めることができます。質問や補足があればお知らせください。 |
| Mpoxについて教えてください。 | Mpox（モンキーポックス）は、モンキーポックスウイルス（MPXV）によって引き起こされるウイルス性の感染症です。このウイルスは、天然痘ウイルスと同じオルソポックスウイルス属に属し、症状も天然痘と類似していますが、一般的には天然痘よりも軽度です。  ### 症状 モンキーポックスの症状は、感染後6〜13日（潜伏期間が5〜21日の範囲内）で現れることが多いです。主な症状は次のとおりです。  1. **発熱**：一般的に感染後の初期段階で現れます。 2. **頭痛** 3. **筋肉痛** 4. **リンパ節の腫れ**：これは、天然痘には見られない特徴的な症状です。 5. **疲労感** 6. **発疹**：発疹は通常、顔面から始まり、体全体に広がります。発疹は水疱や膿疱になり、最終的にはかさぶたになり、2〜4週間で自然に治癒します。  ### 感染経路 モンキーポックスは、主に感染動物（特に霊長類や小型哺乳類）との接触、または感染者の体液、呼吸器分泌物、皮膚病変との直接接触を介して人に感染します。また、感染者と密接に接触することで人から人への感染も可能です。  ### 予防と治療 モンキーポックスに対する特定の治療法はありませんが、症状を軽減するための支持療法が行われます。また、天然痘ワクチンがモンキーポックスに対しても効果があることが知られており、一部の国ではモンキーポックスの感染リスクが高い人々に対して天然痘ワクチンの接種が推奨されています。  ### 流行 モンキーポックスは、主に中央アフリカや西アフリカの一部の国で流行が見られますが、最近では旅行や国際的な接触によって他の地域でも報告されています。  もし特定のトピックについてもっと詳しく知りたいことがあれば教えてください。 |
| Mpoxについて教えてください。 | Mpox（旧称：Monkeypox、モンキーポックス）は、ウイルス性疾患で、主に中央アフリカおよび西アフリカの一部地域で発生しています。Mpoxウイルスは、サルやげっ歯類などの動物から人間に感染することで知られており、人から人への感染も可能です。以下は、Mpoxに関する主な情報です。  ### 症状 Mpoxの症状は、通常、感染から5〜21日後に現れます。症状は次のような段階で進行します。 1. **初期症状**: 発熱、頭痛、筋肉痛、リンパ節の腫れ、倦怠感などの一般的な風邪のような症状が見られます。 2. **発疹**: 数日後に、発疹が顔、手、足などに現れ、身体全体に広がることがあります。発疹は膿疱（膿が溜まったブツブツ）に変わり、最終的にはかさぶたとなって剥がれ落ちます。  ### 感染経路 Mpoxは、感染した動物の咬傷や傷、または血液、体液、皮膚の病変への接触を通じて感染します。人から人への感染は、飛沫感染、直接接触、または汚染された物品（寝具や衣服など）を介して起こります。  ### 診断 Mpoxは、症状や流行地域での滞在歴などに基づいて診断されますが、確定診断にはPCR検査が必要です。この検査では、血液や皮膚病変のサンプルからウイルスDNAを検出します。  ### 治療 現在、Mpoxに特効薬はありませんが、症状を緩和するための治療が行われます。これには、発熱や痛みの管理、感染部位の清潔保持が含まれます。また、重症例には抗ウイルス薬や免疫グロブリンの使用が検討されることがあります。  ### 予防 予防策としては、動物との接触を避けることや、感染者との濃厚接触を避けることが推奨されます。また、Mpoxに対するワクチンも開発されており、一部の国ではリスクの高い人々に接種が行われています。  Mpoxは比較的稀な疾患ですが、感染が確認された場合は、迅速な対応と隔離が重要です。2022年には世界的に症例が増加し、一部の国で公衆衛生上の関心が高まりました。 |
| Mpoxについて教えてください。 | Mpox（旧称：サル痘、Monkeypox）は、モンキーポックスウイルス（Monkeypox virus）によって引き起こされるウイルス性疾患です。この疾患は、主にアフリカの一部地域で発生していましたが、近年、他の地域でも報告が増えています。  ### 特徴と症状 Mpoxは天然痘に似た症状を引き起こしますが、通常は天然痘よりも軽度です。主要な症状には以下が含まれます：  1. **発熱**：通常は高熱が出ます。 2. **発疹**：体のさまざまな部位に発疹が現れます。顔、手のひら、足の裏に特によく見られます。発疹は最初は小さな斑点（マクラ）として現れ、その後膿疱に変わります。 3. **リンパ節の腫れ**：リンパ節が腫れることが特徴的です。 4. **頭痛、筋肉痛、疲労感**：これらの症状も一般的です。  ### 感染経路 Mpoxは動物から人への接触、または感染者との接触を通じて感染します。感染経路には、感染動物の血液、体液、皮膚の病変との直接接触が含まれます。人から人への感染は、飛沫感染や密接な接触を通じて発生しますが、ヒトからヒトへの伝播は比較的稀です。  ### 治療と予防 現在、Mpoxの特異的な治療法は存在しませんが、症状の緩和を目的とした対症療法が行われます。重症例では抗ウイルス薬が使用されることがあります。また、天然痘ワクチンが一定の効果を持つとされており、予防接種が有効です。  ### 現在の状況 2022年以降、世界各地でMpoxの感染が報告され、特に男性間の性的接触を介して感染が広がっているとされています。世界保健機関（WHO）や各国の保健当局は、感染拡大を防ぐための対策を強化しています。  Mpoxに関する詳細な情報は、WHOや各国の公衆衛生機関からの最新のガイダンスを参照することをお勧めします。 |
| Mpoxについて教えてください。 | Mpox（エムポックス）は、以前はサル痘（Monkeypox）として知られていた感染症です。Mpoxは、主に動物（特に霊長類やげっ歯類）から人に感染し、また人から人へも感染するウイルス性の疾患です。この病気は、1970年代に最初に発見され、特にアフリカの中央部および西部で流行していましたが、最近では他の地域でも報告されています。  ### 主な特徴 - **原因ウイルス**: Mpoxウイルスは、ポックスウイルス科に属するオルソポックスウイルス属の一種です。これは、天然痘ウイルスと同じグループに属しますが、重症度は一般的に低いです。 - **症状**: 初期症状は、発熱、頭痛、筋肉痛、リンパ節の腫れ、そして倦怠感などです。その後、発疹が現れます。この発疹は、水疱、膿疱へと進行し、最終的にはかさぶたとなり、治癒します。 - **感染経路**: 動物からの感染は、動物に噛まれる、引っかかれる、または動物の体液や病変に直接触れることによって発生します。人から人への感染は、感染者の体液や発疹に触れること、または飛沫を介して起こります。 - **治療と予防**: 現時点では、特異的な治療法はありませんが、症状の管理が行われます。天然痘ワクチンは、一定の予防効果があるとされています。  ### 公衆衛生の重要性 Mpoxは、特に集団感染が発生した場合に、公共衛生上の懸念となります。感染拡大を防ぐためには、早期診断、感染者の隔離、予防接種、および適切な衛生管理が重要です。  Mpoxに関する理解は進んでおり、研究や情報提供が進められていますが、正確で最新の情報を得ることが重要です。 |
| Mpoxについて教えてください。 | Mpox（モンキーポックス）は、Monkeypoxウイルス（MPXV）によって引き起こされるウイルス性疾患です。この病気は、痘瘡ウイルス（バリオラウイルス）と同じオルソポックスウイルス科に属しています。モンキーポックスは、1958年に研究所で飼育されていたサルで初めて確認されたため、この名前が付けられました。しかし、主な感染源は霊長類ではなく、齧歯類や小型哺乳類が自然宿主と考えられています。  ### 主な特徴 - **感染経路**: モンキーポックスウイルスは、人から人への直接接触、感染した動物との接触、あるいは汚染された物品を通じて感染します。また、飛沫感染や母子間の垂直感染も報告されています。 - **症状**: 潜伏期間は5〜21日で、発熱、頭痛、筋肉痛、リンパ節腫脹などの初期症状に続き、特徴的な発疹が顔や体全体に現れます。この発疹は水疱や膿疱に進展し、最終的にはかさぶたとなって治癒します。 - **重症度**: 症状は通常軽度から中等度で、ほとんどの患者は数週間で自然に回復しますが、免疫不全状態にある人や重篤な基礎疾患を持つ人では、重症化する可能性があります。死亡率は、旧来の痘瘡ウイルスに比べて低いですが、特定の集団では致死的になることもあります。 - **予防**: 以前は、痘瘡ワクチンがモンキーポックスに対する予防効果があるとされていましたが、近年では、特にモンキーポックスに対して開発された新しいワクチンもあります。また、感染拡大を防ぐためには、感染者との接触を避けることが重要です。  ### 現状と対策 2022年に世界中でモンキーポックスの感染例が急増したことがあり、その後もいくつかの地域で感染が報告されています。世界保健機関（WHO）や各国の保健機関は、迅速な対応と情報共有を通じて、感染拡大を抑えるための取り組みを進めています。  この病気についてのさらなる研究や理解が進む中で、感染予防と治療の選択肢も改善されていくことが期待されています。 |
| エムポックスについて教えてください。 | エムポックス（Mpox）は、以前はサル痘（Monkeypox）として知られていた感染症です。エムポックスは、ウイルス性の疾患であり、主に霊長類やげっ歯類に感染しますが、人間にも感染することがあります。  ### エムポックスの特徴 - **原因ウイルス**: エムポックスウイルス（Mpox virus）は、ポックスウイルス科のオルソポックスウイルス属に属しています。 - **感染経路**: 主に感染動物との直接接触や、その体液や病変部位に触れることで感染します。また、飛沫感染や、汚染された物品を介しての感染も報告されています。 - **症状**: エムポックスの症状は、発熱、頭痛、筋肉痛、リンパ節の腫れに続いて、発疹が顔や体に広がります。発疹は最初は斑点状で、次第に膿疱やかさぶたに変わります。病気の進行は一般的に2〜4週間です。 - **重症化リスク**: 特に免疫力が低下している人や、子供、妊婦は重症化するリスクが高いとされています。 - **治療**: 特異的な治療法はありませんが、症状を緩和するための対症療法が行われます。ウイルスに対する免疫グロブリンや抗ウイルス薬が使用されることもあります。 - **予防**: 天然痘ワクチンがエムポックスに対して一定の予防効果があるとされていますが、特定のエムポックスワクチンも開発が進められています。  ### 最近の動向 近年、エムポックスは特定の地域で感染拡大が確認されており、特に西アフリカと中央アフリカでの流行が報告されています。2022年には、世界的にエムポックスの感染者が増加し、各国で公衆衛生対策が強化されました。感染の抑制には、迅速な診断と隔離、接触者追跡が重要です。  エムポックスに関する情報を理解しやすくするためには、信頼性の高い医療機関や公的機関からの情報を参照することが推奨されます。 |
| エムポックスについて教えてください。 | エムポックス（Mpox）は、以前は「サル痘」（Monkeypox）として知られていたウイルス感染症で、主に中央アフリカと西アフリカの特定の地域で発生しています。Mpoxウイルスは、天然痘ウイルスと同じ属に属しており、発疹を伴うことが多い感染症です。ここでは、エムポックスに関する基本的な情報を説明します。  ### エムポックスの特徴: 1. **原因**: Mpoxウイルス（Monkeypox virus）は、オルソポックスウイルス属に属するウイルスです。   2. **感染経路**: エムポックスは、感染した動物（通常は野生のげっ歯類や霊長類）から人に感染することが多いです。ヒトからヒトへの感染もあり、特に接触感染や飛沫感染が一般的です。  3. **症状**:  - 初期症状は、発熱、頭痛、筋肉痛、リンパ節の腫れなどです。  - 数日後、発疹が現れ、体全体に広がることがあります。発疹は水疱に進行し、最終的にかさぶたになります。  - 感染期間は通常2〜4週間です。  4. **治療**: 特定の治療法はないものの、症状に対する対症療法が行われます。軽症で自然治癒する場合も多いですが、重症化することもあり、その際には医療機関での治療が必要です。  5. **予防**: エムポックスに対する特異的なワクチンは存在しませんが、天然痘ワクチンがある程度の防御効果を持っているとされています。また、感染リスクの高い地域への旅行を避けることや、感染が確認された動物との接触を避けることが推奨されます。  エムポックスは、特に近年の国際的な移動の増加に伴い、アフリカ以外の地域でも散発的に発生しています。感染拡大を防ぐためには、早期の診断と適切な隔離が重要です。 |
| エムポックスについて教えてください。 | エムポックス（Mpox）は、以前は「サル痘」として知られていた感染症です。これはポックスウイルス科に属するモンキーポックスウイルス（Monkeypox virus）によって引き起こされます。エムポックスは、人に感染することができる珍しい病気で、主にアフリカの一部地域で発生してきましたが、近年では他の地域でも報告が増加しています。  ### エムポックスの特徴 - **症状**: エムポックスの症状には、発熱、頭痛、筋肉痛、リンパ節の腫れ、そして典型的な皮膚の発疹が含まれます。この発疹は最初に顔に現れ、次第に体全体に広がります。発疹は水疱状で、時間が経つと膿疱になり、その後かさぶたを形成します。 - **感染経路**: エムポックスは、感染した動物（特にげっ歯類や霊長類）との接触、または感染者との密接な接触を通じて人に感染します。ウイルスは、体液、呼吸器分泌物、感染した皮膚の病変との直接接触、あるいはこれらの病変に触れた物品を介して広がることがあります。 - **治療と予防**: エムポックスに対する特定の治療法は存在しませんが、症状は対症療法で管理されます。感染を予防するためには、感染した動物や人との接触を避けることが重要です。また、特定のワクチン（天然痘ワクチン）は、エムポックスに対する防御効果があるとされています。  エムポックスは通常、自然に回復する病気ですが、重症化するケースもあり、特に免疫が低下している人や子供、高齢者においてはリスクが高いとされています。感染が疑われる場合や、エムポックスが流行している地域への渡航を計画している場合には、医療機関での相談が推奨されます。 |
| エムポックスについて教えてください。 | エムポックス（Mpox）は、以前は「サル痘（Monkeypox）」として知られていた感染症です。2022年に世界保健機関（WHO）が、エムポックスという名称に変更しました。この病気は、サル痘ウイルス（Monkeypox virus）によって引き起こされ、天然痘ウイルスと同じウイルス科に属します。  ### エムポックスの特徴 - **感染経路**: エムポックスは、動物から人間に感染する人畜共通感染症です。主な感染源は感染した動物（特に齧歯類や霊長類）で、これらの動物に咬まれる、接触する、または体液に触れることで感染する可能性があります。また、人から人への感染もあり、特に密接な接触によって広がることがあります。   - **症状**: エムポックスの症状は、発熱、頭痛、筋肉痛、倦怠感、リンパ節の腫れなど、初期の症状はインフルエンザに似ています。その後、発疹が現れ、膿疱（うみのたまったぶつぶつ）ができることがあります。発疹は通常、顔や四肢から始まり、全身に広がることがあります。  - **潜伏期間**: 一般的に、エムポックスの潜伏期間は6日から13日ですが、場合によっては5日から21日までの範囲があります。  - **予防と治療**: エムポックスに対しては、天然痘のワクチンがある程度の予防効果を持つとされています。また、抗ウイルス薬や免疫グロブリン療法が治療に用いられることがあります。  ### エムポックスの歴史 エムポックスは1970年に最初にヒトで確認され、その後アフリカの中部や西部で発生が報告されました。しかし、2022年以降、アフリカ以外の地域でも感染が広がり、国際的な注目を集めるようになりました。  エムポックスは一般的に致死率が低いですが、重篤なケースや合併症が発生することがあり、特に免疫不全者や小児、高齢者にはリスクが高まります。  このように、エムポックスは人畜共通感染症として注目されていますが、予防策や治療法の進展によって、その影響を最小限に抑えることが可能です。 |
| エムポックスについて教えてください。 | エムポックス（Mpox）は、以前は「サル痘（Monkeypox）」として知られていたウイルス感染症です。この病気は、モンキー痘ウイルスというウイルスによって引き起こされます。エムポックスは、天然痘ウイルスに似ていますが、症状は通常、天然痘よりも軽度です。  ### エムポックスの特徴と症状 エムポックスの感染者は、発熱、頭痛、筋肉痛、倦怠感などの初期症状を経験することがあります。これらの症状の後に、皮膚に発疹が現れ、その後に膿疱（膿がたまった水疱）に発展することがあります。発疹は顔から始まり、全身に広がることが一般的です。  ### 伝染経路 エムポックスは、感染した動物や人との接触、または感染者の体液、発疹に触れることによって人に感染します。また、感染者の咳やくしゃみによる飛沫感染も考えられます。  ### 予防と治療 エムポックスに対する特異的な治療法はありませんが、症状を軽減するための対症療法が行われます。また、天然痘ワクチンはエムポックスに対しても一定の予防効果があるとされています。  エムポックスは、特定の地域に限られて発生することが多いですが、近年では国際的な移動の増加に伴い、他の地域でも感染が報告されています。病気の拡大を防ぐためには、早期発見と適切な管理が重要です。  他に具体的な質問や詳細が必要であれば教えてください。 |
| エムポックスについて教えてください。 | エムポックス（Mpox、旧称モンキーポックス）は、オルソポックスウイルス科に属するモンキーポックスウイルスによって引き起こされる感染症です。エムポックスは、天然痘と類似した症状を引き起こしますが、一般的には天然痘よりも軽度です。  ### 主な特徴と症状 エムポックスの主な症状には以下が含まれます： - **発熱**：感染後5日から21日以内に発熱が見られることがあります。 - **発疹**：顔や体の他の部分に小さな水疱や膿疱が現れることがあります。 - **リンパ節の腫れ**：リンパ節が腫れることがあります。 - **頭痛、筋肉痛、倦怠感**：これらの全身症状も見られることがあります。  ### 伝播経路 エムポックスは、感染した動物や人との接触を通じて広がります。具体的には、動物（特に齧歯類や霊長類）の咬傷、皮膚や粘膜との直接接触、あるいは体液を介して伝染することが知られています。また、感染者との密接な接触や、感染者の体液に触れることでも人から人への感染が起こることがあります。  ### 予防と治療 エムポックスの予防には、感染した動物や人との接触を避けることが重要です。また、天然痘ワクチンがエムポックスに対する一定の予防効果を持つことが示されています。治療には、主に症状の管理が行われ、抗ウイルス薬が使用されることもあります。  エムポックスは、2022年以降、複数の国で流行が確認され、世界的な関心を集めるようになりました。感染拡大を抑えるために、公衆衛生上の対策が強化されています。 |
| エムポックスについて教えてください。 | エムポックス（Mpox）は、以前は「サル痘（Monkeypox）」として知られていたウイルス感染症で、オルソポックスウイルス属に属するウイルスによって引き起こされます。このウイルスは、天然痘ウイルスと同じウイルス属に属していますが、天然痘とは異なり、エムポックスの症状や致死率は比較的軽度です。  ### エムポックスの主な特徴 - **症状**: エムポックスの典型的な症状には、発熱、頭痛、筋肉痛、リンパ節の腫れ、疲労感、そして特徴的な発疹が含まれます。発疹はしばしば顔に始まり、その後、体の他の部分（特に手のひらや足の裏）に広がります。発疹は、斑点状、丘疹、膿疱、かさぶたの形をとり、最終的に治癒します。 - **伝播**: エムポックスは、動物から人間に、または感染者との接触や体液の交換、または感染者が使用した物品を通じて人間に伝播します。主にアフリカの一部の地域でエムポックスが発生しますが、近年では世界の他の地域でも報告されています。 - **予防と治療**: エムポックスに対する特異的な治療法はありませんが、症状を緩和するための対症療法が行われます。天然痘ワクチンがエムポックスに対して一定の効果があるとされています。  2022年には、エムポックスが多くの国で発生し、特に男性間の性的接触によって感染が拡大しました。そのため、世界保健機関（WHO）は、エムポックスに対する監視と対応を強化し、ワクチン接種の推奨などを行っています。  エムポックスについてさらに詳細を知りたい場合や、特定の側面に関心がある場合は教えてください。 |
| エムポックスについて教えてください。 | エムポックス（Mpox）は、以前はサル痘（Monkeypox）として知られていたウイルス感染症です。このウイルスは、ポックスウイルス科に属するオルソポックスウイルス属の一種で、天然痘ウイルスに近縁です。エムポックスは、主に中央および西アフリカの熱帯雨林地域で発生しますが、最近では世界各地で症例が報告されています。  ### 感染経路 エムポックスは、動物から人間への接触、または人から人への接触を通じて感染します。感染源としては、感染した動物（特にげっ歯類や霊長類）の体液や血液、またはウイルスに汚染された物品があります。また、人から人への感染は、主に感染者の皮膚病変や体液、または呼吸器からの飛沫を通じて伝播します。  ### 症状 エムポックスの症状は、通常、発熱、頭痛、筋肉痛、疲労感などのインフルエンザ様症状で始まり、その後に発疹が出現します。発疹は、顔面から始まり、全身に広がることが多いです。発疹は水疱や膿疱に進行し、最終的にかさぶたとなって治癒します。症状は通常2〜4週間続きますが、免疫が低下している場合や幼児では重症化することがあります。  ### 治療と予防 エムポックスには特異的な治療法はありませんが、症状を緩和するための対症療法が行われます。重症例や合併症がある場合には、抗ウイルス薬が使用されることがあります。予防には、感染源との接触を避けることが最も効果的です。また、サル痘に対する予防接種も一部の地域で行われており、特に感染リスクの高い人々に推奨されています。  ### 現在の状況 2022年以降、エムポックスの世界的な感染拡大が注目されています。この拡大は、従来の流行地域外での症例が増加していることを特徴としています。公衆衛生当局は、感染拡大を抑制するための監視や対応策を強化しています。  この情報は、エムポックスに関する基本的な理解を深めるためのものであり、最新の情報については公衆衛生当局や医療機関の公式発表を参考にしてください。 |
| エムポックスについて教えてください。 | エムポックス（Mpox）は、以前はサル痘（Monkeypox）と呼ばれていた病気で、主にウイルスによって引き起こされます。この病気は、人間や動物に感染する可能性があり、特にサルやげっ歯類などの動物から人間に感染することが知られています。エムポックスは、天然痘ウイルスと同じウイルス科に属するモンキーポックスウイルス（Monkeypox virus）によって引き起こされます。  ### 症状 エムポックスの症状は、発熱、頭痛、筋肉痛、疲労感、リンパ節の腫れなど、インフルエンザのような症状で始まります。その後、発疹が現れ、皮膚に水疱や膿疱ができることがあります。発疹は、通常、顔や手足から始まり、全身に広がることが多いです。  ### 感染経路 エムポックスは、感染した動物との接触や、その動物にかまれたり、引っかかれたりすることで感染することが多いです。また、感染者の体液や発疹に直接触れることでも感染が広がる可能性があります。  ### 治療と予防 エムポックスには特定の治療法はなく、症状を緩和するための支持療法が行われることが一般的です。予防としては、感染した動物や感染者との接触を避けることが重要です。また、サル痘ワクチンはエムポックスに対してある程度の予防効果があるとされています。  ### 最近の状況 2022年から2023年にかけて、エムポックスは複数の国で発生が報告され、特に都市部での感染拡大が懸念されています。このため、エムポックスに対する注意喚起や予防対策が強化されています。  エムポックスについてさらに詳細な情報が必要であれば、具体的な質問にお答えできます。 |
| エムポックスについて教えてください。 | エムポックス（Mpox）は、旧称「サル痘（Monkeypox）」として知られるウイルス感染症です。2022年に世界保健機関（WHO）が、差別や誤解を避けるために「Mpox（エムポックス）」という新しい名称を推奨しました。この病気は、特に西アフリカと中央アフリカの特定の地域で流行していますが、近年は他の地域でも報告されています。  ### エムポックスの原因 エムポックスは、サル痘ウイルス（Monkeypox virus）によって引き起こされます。このウイルスは、オルソポックスウイルス属に属し、天然痘ウイルスと密接に関連しています。人間だけでなく、動物（特に霊長類やげっ歯類）にも感染することがあります。  ### 感染経路 エムポックスは、感染した動物や人との接触を通じて広がります。感染経路としては、以下のようなものが考えられます。 - **動物から人へ**: 感染した動物に咬まれたり、引っかかれたり、体液や血液に触れることで感染します。 - **人から人へ**: 感染者との密接な接触（皮膚の傷口や粘膜への接触）、または感染者の呼吸器分泌物や体液との接触によって感染することがあります。  ### 症状 エムポックスの症状は、感染から6～13日後に現れることが多いですが、5～21日後に発症することもあります。主な症状は次のとおりです。 - **発熱** - **頭痛** - **筋肉痛** - **疲労感** - **リンパ節の腫れ** - **発疹**: 発疹は顔から始まり、体全体に広がることがあります。発疹は水疱や膿疱を形成し、最終的にはかさぶたとなります。  ### 予防と治療 エムポックスに対する特効薬はありませんが、症状は一般的に自然に治ります。治療には、症状を和らげるための支持療法が用いられます。予防には、感染リスクのある地域への不要な旅行を避け、感染が疑われる場合は医療機関に相談することが重要です。  また、天然痘ワクチンがエムポックスに対してもある程度の予防効果があるとされています。天然痘ワクチン接種は、特に高リスクの人々に対して推奨されることがあります。  エムポックスは、近年、特に世界的な健康問題として注目されており、各国での監視と対応が進められています。 |
| サル痘について教えてください。 | サル痘（Monkeypox）は、ウイルス性の感染症で、サル痘ウイルスによって引き起こされます。サル痘は、特にアフリカの一部地域で見られる珍しい病気で、天然痘に似た症状を引き起こしますが、通常は天然痘よりも軽度です。近年、アフリカ以外の地域でもサル痘の感染が確認されることがあり、国際的な関心が高まっています。  ### 症状 サル痘の症状は、感染から約5〜21日の潜伏期間を経て現れます。主な症状は以下の通りです： - 発熱 - 頭痛 - 筋肉痛 - 疲労感 - リンパ節の腫れ  その後、皮膚に発疹が現れ、発疹は水疱や膿疱に進展し、最後にはかさぶたとなって治癒します。発疹は顔や手のひら、足の裏などに広がることが多く、体の他の部位にも影響を及ぼすことがあります。  ### 感染経路 サル痘ウイルスは、感染した動物（特にサルやげっ歯類）との直接接触や、感染した人との密接な接触を通じて感染します。特に皮膚の傷や粘膜を通じてウイルスが体内に侵入することで感染が広がります。  ### 予防と治療 サル痘に対する特定の治療法はありませんが、症状を軽減するための対症療法が行われます。また、天然痘ワクチンがサル痘に対してある程度の効果があるとされています。感染予防のためには、感染者との接触を避けることや、感染が疑われる場合には医療機関に相談することが重要です。  サル痘のアウトブレイクは、通常は迅速に管理され、広範な流行には至らないことが多いですが、注意が必要な感染症です。 |
| サル痘について教えてください。 | サル痘（さるとう、Monkeypox）は、サル痘ウイルスによって引き起こされるウイルス性の疾患です。サル痘は天然痘と同じオルソポックスウイルス属に属しており、症状や感染経路に類似点がありますが、サル痘は通常、天然痘よりも軽度です。  ### サル痘の主な特徴  1. **感染経路**:  - サル痘ウイルスは、感染した動物（主に小型哺乳類や霊長類）との接触、または感染者との密接な接触によって人に感染します。  - ウイルスは皮膚の病変、体液、または呼吸器の飛沫を介して広がることがあります。  2. **症状**:  - 発熱、頭痛、筋肉痛、リンパ節の腫れなど、全身症状が初期に現れます。  - その後、顔や体に発疹が出現し、水疱や膿疱へと進行します。  - 症状は通常、2～4週間持続します。  3. **治療**:  - 特異的な治療法はありませんが、対症療法や感染管理が行われます。  - 天然痘ワクチンがサル痘に対してある程度の予防効果を持つとされています。  4. **予防**:  - 感染者や感染が疑われる動物との接触を避けることが重要です。  - 感染が疑われる場合には、隔離と感染予防措置が推奨されます。  ### 発生状況 サル痘は主に中央アフリカと西アフリカの一部地域で風土病として発生していますが、近年では他の地域でも散発的なアウトブレイクが報告されています。2022年には、これまでサル痘があまり見られなかった地域でも複数の感染事例が確認され、国際的な公衆衛生上の関心が高まりました。  サル痘は通常、重篤な合併症を引き起こすことは少ないですが、特に免疫が低下している人や子供、高齢者では重症化する可能性があります。 |
| サル痘について教えてください。 | サル痘（サル痘ウイルス感染症）は、サル痘ウイルスによって引き起こされるウイルス感染症です。サル痘はもともとアフリカ中央部および西部の一部地域で発見され、ヒトに感染する場合もありますが、動物からヒトへ、あるいはヒトからヒトへの感染が確認されています。  ### 主な症状 サル痘の症状は、天然痘に似ていますが、通常は軽度です。感染後、潜伏期間は一般に6～13日ですが、5～21日の範囲で発症することもあります。主な症状には以下が含まれます： - 発熱 - 頭痛 - 筋肉痛 - 背中の痛み - リンパ節の腫れ - 倦怠感  発熱から1～3日後には発疹が現れ、顔から始まって体全体に広がることが多いです。発疹は数日から数週間かけて膿疱や水疱に発展し、最終的にはかさぶたを形成します。  ### 感染経路 サル痘は、感染した動物（特にサルや齧歯類）との接触や、感染者の体液や発疹に触れること、または飛沫感染によって広がることがあります。感染者との直接接触や汚染された物品との接触も感染リスクがあります。  ### 治療と予防 サル痘に対する特定の治療法はありませんが、症状に応じた対症療法が行われます。また、天然痘ワクチンがサル痘に対して一定の予防効果があることが知られています。感染拡大を防ぐためには、感染者の隔離、感染者との接触を避けること、そして予防接種が重要です。  ### 公衆衛生上の重要性 サル痘は天然痘が根絶された後も、まれに発生する感染症であり、特にアフリカでの発生が注目されています。2022年には、アフリカ以外の地域でも感染例が報告され、国際的な関心が高まっています。  何か他にサル痘について詳しく知りたいことがあれば教えてください。 |
| サル痘について教えてください。 | サル痘（サルとう、Monkeypox）は、ウイルス性の感染症で、サル痘ウイルス（Monkeypox virus）によって引き起こされます。このウイルスは、ポックスウイルス科に属し、天然痘ウイルスと同じグループに属しています。サル痘は、1950年代にアフリカのカニクイザルで初めて確認され、人間にも感染することが確認されています。  ### 特徴と症状 サル痘は、以下のような特徴と症状を持ちます： - **初期症状**: 発熱、頭痛、筋肉痛、疲労感、リンパ節の腫れなど、インフルエンザに似た症状が現れます。 - **皮膚症状**: 1～3日後に、顔を中心に発疹が現れ、その後、体の他の部分にも広がります。発疹は、水疱や膿疱に進行し、最終的にはかさぶたになり、治癒します。 - **潜伏期間**: 通常、7～14日ですが、最大21日間続くこともあります。  ### 感染経路 サル痘ウイルスは、人間や動物からの感染が可能です。以下のような経路で感染が広がります： - **動物から人間へ**: 主に動物の血液、体液、皮膚の病変などに触れることによって感染します。感染源としては、感染した霊長類や齧歯類（げっしるい）などが考えられます。 - **人間から人間へ**: 感染者の皮膚病変や体液、呼吸器分泌物との接触、または飛沫感染によって広がることがあります。  ### 予防と治療 サル痘に対する特定の治療法はありませんが、症状を緩和するための支持療法が行われます。天然痘ワクチンがサル痘の予防にも効果があるとされており、感染リスクの高い人々には天然痘ワクチンが推奨される場合があります。  ### 流行状況 サル痘は、アフリカ中部および西部の一部地域で風土病として存在していましたが、近年は他の地域でも散発的に発生が報告されています。2022年には、世界各地でサル痘の感染例が急増し、世界的な健康問題となりました。  このように、サル痘は天然痘に似た症状を持ち、感染経路や予防法も共通点がありますが、天然痘とは異なり、感染の拡大は比較的緩やかです。 |
| サル痘について教えてください。 | サル痘（サルとう）は、サル痘ウイルスによって引き起こされる感染症です。サル痘ウイルスは、ポックスウイルス科に属するウイルスで、天然痘ウイルスと似た特徴を持っています。サル痘は主に中央アフリカおよび西アフリカの熱帯雨林地域に分布しており、そこでは野生動物から人に感染することが確認されています。特に齧歯類や霊長類が感染源となることが多いです。  ### 主な特徴 - **症状**: サル痘の症状は、発熱、頭痛、筋肉痛、リンパ節腫脹（リンパ節の腫れ）から始まり、その後、発疹が顔から体全体に広がります。発疹は水疱や膿疱に進行し、最後にはかさぶたとなって治癒します。   - **感染経路**: サル痘は、感染した動物の血液や体液、皮膚や粘膜に直接接触することで人に感染するほか、感染した人との密接な接触や、飛沫感染によっても広がることがあります。  - **重症度**: サル痘の致死率は通常1%から10%とされており、特に若年者や免疫力が低下している人々が重症化しやすいです。ただし、致死率は地域や医療環境によって異なります。  - **予防と治療**: 現在、サル痘に対する特異的な治療薬はありませんが、天然痘ワクチンがサル痘に対してもある程度の効果があるとされています。また、感染が確認された場合には隔離措置を講じ、症状に応じた支持療法を行います。  ### 近年の状況 2022年には、アフリカ以外の地域でもサル痘の感染者が確認され、国際的な関心が高まりました。この発生は、旅行者を介したウイルスの拡散や、人々の間での二次感染によるものと考えられています。  サル痘の感染が疑われる場合や発生地域に旅行する際には、感染予防策をしっかりと講じることが重要です。 |
| サル痘について教えてください。 | サル痘（Monkeypox）は、ポックスウイルス科に属するウイルスによって引き起こされる感染症です。サル痘ウイルスは、人間や動物に感染する可能性があり、特にサルやげっ歯類でよく見られますが、ヒトにも感染することがあります。  ### 症状 サル痘の症状は、発熱、頭痛、筋肉痛、リンパ節の腫れなどのインフルエンザに似た症状から始まり、その後、発疹が体に現れることが特徴です。発疹は、顔や体に広がり、最終的には水疱や膿疱に進行し、かさぶたになります。これらの皮膚症状は非常に特徴的であり、診断の手がかりとなります。  ### 伝播経路 サル痘ウイルスは、感染動物との直接接触、動物由来の食品や物品を介してヒトに感染することがあります。また、感染者の体液や呼吸器からの飛沫を介してヒトからヒトへも伝播することがあります。  ### リスクと予防 サル痘は通常、比較的軽度な病気とされていますが、重症化するケースもあります。特に免疫力が低下している人や幼児、高齢者などは、重症化しやすい傾向があります。予防のためには、感染の疑いがある動物や人との接触を避け、衛生管理を徹底することが重要です。  ### 治療 サル痘には特効薬はありませんが、対症療法が行われます。患者には安静、適切な水分補給、皮膚の清潔を保つことが推奨されます。また、サル痘ウイルスは天然痘ウイルスと非常に近いため、天然痘ワクチンがサル痘の予防や症状軽減に有効であるとされています。  サル痘は、西アフリカや中央アフリカの特定地域でよく見られますが、最近ではその他の地域でも報告されています。国際的な移動や貿易の増加に伴い、感染拡大のリスクが高まっていることが懸念されています。 |
| サル痘について教えてください。 | サル痘（Monkeypox）は、サル痘ウイルス（Monkeypox virus）によって引き起こされるウイルス感染症です。この病気は天然痘と同じオルトポックスウイルス属に属しますが、天然痘よりも症状が軽いとされています。サル痘は主にアフリカの中央部および西部の熱帯雨林地域で発生しますが、近年ではアフリカ以外の地域でも感染が確認されています。  ### 症状 サル痘の主な症状は、以下のようなものがあります： - 発熱 - 頭痛 - 筋肉痛 - 倦怠感 - リンパ節の腫れ - 発疹（顔や体全体に広がる水疱や膿疱）  発疹は最初に顔に現れ、その後、体や四肢に広がります。発疹は数日から数週間続き、その後、かさぶたとなって治癒します。  ### 感染経路 サル痘は、感染した動物（特に霊長類や小型哺乳類）との接触、または感染者との密接な接触により伝播します。ウイルスは傷口、呼吸器、または粘膜を通じて体内に侵入します。人から人への感染も可能ですが、感染力は比較的低いです。  ### 治療 サル痘に特異的な治療法はありませんが、症状を緩和するための対症療法が行われます。重症例では、抗ウイルス薬や免疫グロブリンの投与が考慮されることもあります。  ### 予防 サル痘の予防には、感染した動物や人との接触を避けることが重要です。また、天然痘ワクチンがサル痘に対してもある程度の効果があるとされています。  サル痘は一般的には軽症で自然に治癒しますが、場合によっては重症化することもあるため、感染が疑われる場合は早めに医療機関を受診することが推奨されます。 |
| サル痘について教えてください。 | サル痘（モンキーポックス、Mpox）は、ウイルス感染症で、サル痘ウイルス（Monkeypox virus, MPXV）によって引き起こされます。このウイルスは、天然痘と同じウイルス科に属しており、天然痘ウイルスとは近縁です。しかし、サル痘は天然痘よりも軽度の症状を引き起こし、致死率も低いとされています。  ### サル痘の症状 サル痘の主な症状は以下の通りです： - 発熱 - 頭痛 - 筋肉痛 - リンパ節の腫れ - 疲労感 - 発疹：感染から数日後に顔から始まり、体全体に広がることが多い。発疹は水疱、膿疱、かさぶたの順に変化します。  症状は通常2〜4週間続きますが、ほとんどの患者は自然に回復します。ただし、免疫力が低下している人や子供など、一部の人々では重症化する可能性があります。  ### 感染経路 サル痘ウイルスは、以下のような経路で人に感染します： - 感染動物（通常は齧歯類や霊長類）からの接触 - 感染者との直接接触や体液、皮膚の傷からの感染 - 感染者の衣類や寝具などの接触  ### 予防と治療 サル痘には特定の治療法はないものの、対症療法が行われます。ワクチン接種は予防に有効であり、特に天然痘ワクチンはサル痘に対しても一定の効果があるとされています。感染予防のためには、感染が疑われる動物や人との接触を避け、感染拡大を防ぐための衛生管理が重要です。  ### 流行状況 サル痘はもともと中央アフリカや西アフリカで発生していましたが、近年では他の地域でも確認されるようになり、2022年には世界的に注目を集めました。  何か特定のトピックや詳細についてお知りになりたいことがあれば教えてください。 |
| サル痘について教えてください。 | サル痘（サル痘ウイルス感染症）は、サル痘ウイルスによって引き起こされる希少なウイルス感染症です。サル痘ウイルスは、天然痘ウイルスと同じオルソポックスウイルス属に属しており、天然痘と似た症状を引き起こしますが、通常は天然痘よりも重症度は低いとされています。  ### 主な特徴と症状 - **発症**: サル痘の潜伏期間は通常6～13日ですが、5～21日までの範囲で変動することがあります。発症は発熱、頭痛、筋肉痛、リンパ節腫脹、寒気、倦怠感などの一般的な症状から始まります。 - **発疹**: 発症から1～3日後に皮膚に発疹が現れます。発疹は、顔から始まり、次第に体の他の部分（特に手のひらや足の裏）に広がります。発疹は斑点から丘疹、膿疱、かさぶたへと進行します。 - **伝染性**: サル痘ウイルスは、感染した動物（特に霊長類や齧歯類）から人に感染することが多く、人から人への感染も可能です。感染経路としては、体液や皮膚病変との直接接触、飛沫感染、または汚染された物品（例：寝具や衣類）を介した接触が考えられます。  ### 発生地域とリスク サル痘は主に中央アフリカと西アフリカの一部の地域で発生していますが、近年では他の地域でも報告されています。感染リスクが高いのは、これらのエンドミック地域に住んでいるか、これらの地域を訪れた人々です。  ### 予防と治療 - **予防**: サル痘の予防には、感染者との接触を避けること、感染した動物と接触しないことが重要です。また、天然痘ワクチンがサル痘に対して一定の効果があるとされていますが、予防接種はリスクのある人々に限定されています。 - **治療**: サル痘の特異的な治療法は存在しませんが、症状に応じた対症療法が行われます。重症例や合併症がある場合は、入院が必要になることがあります。  サル痘は、現在のところ非常にまれな疾患ですが、ウイルスの感染が拡大するリスクがあるため、発生地域における監視と対策が重要です。 |
| サル痘について教えてください。 | サル痘（さるとう、英: Monkeypox）は、ウイルス性の感染症で、主に中央および西アフリカの地域で発生しています。病原体はサル痘ウイルス（Monkeypox virus）で、これは天然痘ウイルスと同じオルソポックスウイルス属に属しますが、天然痘ほど重篤なものではありません。  ### サル痘の特徴と症状  サル痘の症状は、天然痘に似ており、以下のような特徴があります： - **潜伏期間**：通常、ウイルスに感染してから6～13日（最大21日）で発症します。 - **初期症状**：発熱、頭痛、筋肉痛、疲労感など、インフルエンザに似た症状が現れます。また、リンパ節の腫れも見られることが特徴です。 - **発疹**：発症後1～3日後に発疹が現れ、顔、手足、体幹に広がります。発疹は水疱や膿疱に進行し、かさぶたになって最終的に落ちます。  ### 伝播経路  サル痘は主に動物（特にサル、齧歯類など）から人間に感染するズーノーシス（動物由来感染症）です。人間同士の感染も起こり得ますが、通常は密接な接触が必要です。感染は、動物の血液、体液、皮膚や粘膜の傷口に触れることで広がります。また、飛沫感染や、感染者の皮膚病変との直接接触によっても伝播することがあります。  ### 予防と治療  サル痘の予防には、感染した動物や人との接触を避けることが重要です。天然痘のワクチンは、サル痘に対してもある程度の予防効果がありますが、専用のサル痘ワクチンも開発されています。治療法としては、主に対症療法が行われ、特異的な抗ウイルス薬も一部使用されることがあります。  サル痘は、一般に軽度の経過をたどりますが、重症化する場合もあり、特に免疫力が低下している人や子供ではリスクが高くなります。  サル痘についてさらに詳しい情報が必要でしたら、お知らせください。 |
